# Effects of treadmill running on extracellular basal levels of glutamate and GABA at dentate gyrus of streptozotocin-induced diabetic rats

**Published:** 2010

**Authors:** Parham Reisi, Hojjatallah Alaei, Shirin Babri, Mohammad Reza Sharifi, Gisue Mohaddes, Elaheh Soleimannejad, Bahman Rashidi

**Affiliations:** a Department of Physiology, School of Medicine, Isfahan University of Medical Sciences, Isfahan, Iran; b Neurosciences Research Center, Tabriz University of Medical Sciences, Tabriz, Iran; c Department of Physiology, School of Medicine, Tabriz University of Medical Sciences, Tabriz, Iran; d Department of Physiology, Pasteur Institute of Iran, Tehran, Iran; e Department of Anatomy and Histology, School of Medicine, Isfahan University of Medical Sciences, Isfahan, Iran

**Keywords:** Diabetes, Glutamate, GABA, Dentate Gyrus, Treadmill Running, Microdialysis, Hippocampus, Streptozotocin

## Abstract

**BACKGROUND:**

The present study evaluated the effects of treadmill running on extracellular basal levels of glutamate and GABA at dentate gyrus of streptozotocin-induced diabetic rats.

**METHODS:**

After 12 weeks of diabetes induction and exercise period, extracellular levels of glutamate and GABA were investigated.

**RESULTS:**

The results showed that glutamate levels were significantly decreased in diabetes-rest group comparing to the control-rest and the diabetes-exercise groups.

**CONCLUSIONS:**

The findings support the possibility that treadmill running is helpful in alleviating neurotransmitter homeostasis and alterations in transmission in diabetes mellitus.

Although diabetes mellitus impairs learning and memory,^1^ the mechanism of these impairments is not been well understood and treatment with insulin reversed them partially.^2^ It has been demonstrated that diabetes affects synthesis and release of neurotransmitters that are involved in learning and memory in hippocampus such as glutamate.^3^

Due to the fact that regular physical exercise has beneficial effects on neural health and function in diabetes,^4^–^6^ the main objective of this study was to determine the effects of streptozotocin-induced diabetes and treadmill running on basal levels of glutamate and GABA in dentate gyrus of rats.

## Methods

The subjects were male wistar rats (230 ± 20 g) that were divided into four groups (n = 6-7): the control-rest, the control-exercise, the diabetes-rest and the diabetes-exercise. To induce diabetes, streptozotocin (60 mg/kg in saline i.p.) was given to each animal. Animals with blood glucose levels higher than 300 mg/dl after 3 days were selected.^5^ Exercise protocol was daily treadmill running at a speed of 17 m/min for 40 minutes for 12 weeks at 0° of inclination.

After 12 weeks of diabetes induction and exercise duration, rats were anesthetized with urethane (1.8 g/kg i.p.)^5^ and through stereo-taxic surgery a microdialysis probe (dialysis membrane of 1 mm in length) was placed in the dentate gyrus and using a microdialysis pump the probes were perfused with artificial cerebrospinal fluid at a rate of 2 μl/min and the dialysates were collected for 1 hour.

Measurement was made by reverse-phase high pressure liquid chromatography coupled to fluorescence detection, following pre-column derivatization with o-phthaladialdehyde.^3^

Data were analyzed statistically using oneway ANOVA followed by Tukey’s test. The significant level was set at p < 0.05. Results are expressed as mean ± SEM.

## Results

The blood glucose concentrations at the end of experiments are shown in table 1.

**Table 1:** Blood glucose concentrations at the end of experiments

Group	Control		Diabetes	
Condition	Rest	Exercise	Rest	Exercise
Blood glucose (mg/dl)	85 ± 7	88.2 ± 11.4 †	≥ 600 *†	481 ± 34.7*

* Significantly different (p < 0.05) from the control-rest group

† Significantly different (p < 0.05) from the diabetes-exercise group

**Figure 1:**
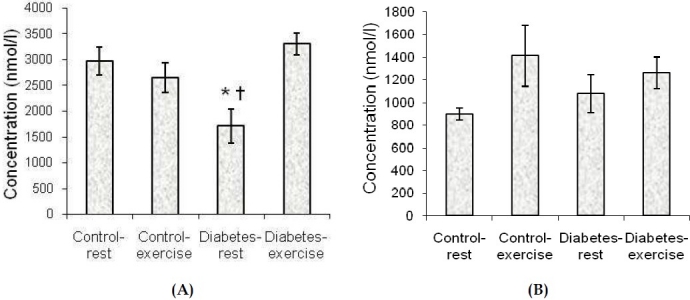
Effects of STZ-induced diabetes and exercise on the extracellular glutamate (a) and GABA (b) content of the microdialysate from the dentate gyrus of anesthetized rats after 12 weeks of diabetes induction and exercise duration. Data are expressed as mean ± SEM. (n = 6-7) * P < 0.05 with respect to the control group † P < 0.05 with respect to the diabetes-exercise group

As it is shown in figure 1, glutamate levels were significantly decreased in diabetes-rest group comparing to the control-rest (p < 0.05) and the diabetes-exercise groups (p < 0.05); however, there were no significant differences between the other groups. In addition GABA levels had no significant differences between the groups.

## Discussion

This study reported that diabetes reduces basal levels of dentate gyrus glutamate but not GABA, and that impairments are attenuated following exercise.

Although exercise seems to have both preventive and therapeutic effects on the defects of brain functions in diabetes, the underlying mechanisms are poorly understood. Possible explanations are:

Exercise prevents suppression of cell proliferation that is produced by diabetes in the dentate gyrus.^4^

In diabetes, hyperglycemia decreases extracellular gulutamate^7^ ; however studies have demonstrated that exercise lowers hyperglycemia (Table 1).^8^

Exercise increases the expression of hippo-campal neurotrophic factors^9^ that are decreased in diabetes.^10^

## Conclusions

The data correspond to the possibility that treadmill running is helpful in alleviating neurotransmitter homeostasis and alterations in transmission in diabetes mellitus.
